# Effect of Nonprotein Components for Lipid Oxidation in Emulsions Stabilized by Plant Protein Extracts

**DOI:** 10.1021/acsfoodscitech.3c00691

**Published:** 2024-03-22

**Authors:** Katharina Münch, Simeon Stoyanov, Karin Schroën, Claire Berton-Carabin

**Affiliations:** † Laboratory of Food Process Engineering, Wageningen University & Research, Bornse Weilanden 9, 6708 WG Wageningen, The Netherlands; ‡ Laboratory of Physical Chemistry and Soft Matter, Wageningen University & Research, Stippeneng 4, 6708 WE Wageningen, The Netherlands; § Singapore Institute of Technology, 10 Dover Drive, 138683 Singapore, Singapore; ∥ INRAE, UR BIA, 44300 Nantes, France

**Keywords:** lipid oxidation, plant protein ingredients, non-protein components, pea protein, soy protein, emulsion

## Abstract

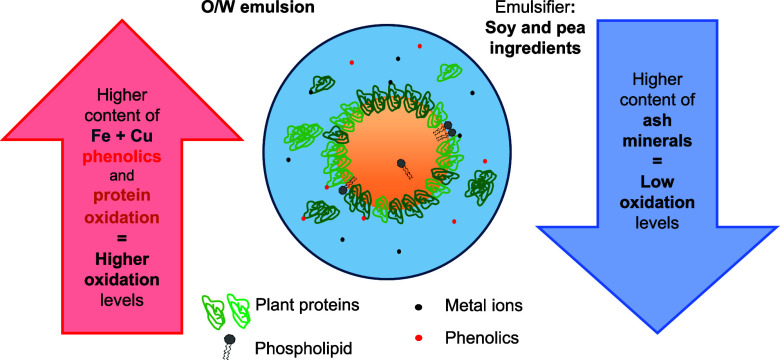

Plant protein ingredients are rich in non-protein components of which the antioxidant and pro-oxidant effects are expected to be considerable. In this paper, commercial soy and pea protein isolates and concentrates were selected by using their soluble fractions to prepare oil-in-water (O/W) emulsions. Emulsions stabilized with soy protein isolates were more prone to lipid oxidation than those with soy protein concentrate or pea protein isolate. Compositional analysis revealed that the soluble fraction of soy protein isolates contained higher concentrations of phenolic compounds and metals (iron and copper) but lower mineral and ash contents than those of soy protein concentrate and pea protein isolate. Correlating the composition to oxidation in emulsions highlighted the significant role of non-protein components, alongside the protein’s oxidative state. These findings are relevant for the use of alternative proteins in food formulation, a practice often promoted as sustainable yet that may come with repercussions for oxidative stability.

## 1 Introduction

Food emulsions are prone to physical and oxidative destabilization and therefore need to be appropriately protected. For physical stability, interface coverage of the droplets by suitable emulsifiers is needed to prevent, e.g., droplet coalescence and flocculation. For sustainability reasons, a lot of attention currently goes to the use of plant-based ingredients as alternatives for animal-based ingredients in food emulsion design.^1^ The oxidative stability of emulsions is related to the prevention of deterioration of polyunsaturated lipids through oxidation, which is a major concern for food quality.^2^ It is often combatted by the addition of synthetic antioxidants (e.g., EDTA), and with natural antioxidants^3,4^ due to legislation constraints and consumer preference for clean-labeled products.

Proteins can act as natural antioxidants through their ability to scavenge free radicals or chelate metal ions.^5^ This has been extensively studied and exemplified with animal-derived proteins, such as dairy proteins (whey proteins and caseins). When plant protein ingredients are used as alternative emulsifiers, it has been shown that this can lead to emulsions having a better oxidative stability than references stabilized by animal proteins.^6−8^ In a recent review, we focused on how plant protein ingredients may modulate lipid oxidation in emulsions, and more specifically on the potential influence of the protein fractionation process and of the environmental conditions applied (pH, ionic strength).^9^ We concluded that it is highly challenging to ascribe lipid oxidation in emulsions to specific components present as the ingredient composition depends on the crop and cultivar as well as on the fractionation process.^9^ Besides proteins, that generally account for 50–80 wt % of the ingredient, plant protein concentrates and isolates still contain substantial amounts of non-protein components such as lipids, phytic acid or polyphenols.^10^ Depending on their localization and concentration, these components may affect lipid oxidation by acting as pro- or antioxidants.^9^ However, the detailed composition of plant protein ingredients, in particular regarding the non-protein component part, is generally not determined nor reported in literature. This, associated with the inherent compositional complexity of these ingredients, makes it very difficult to specifically assess the contribution of such non-protein components to lipid oxidation.

This study aimed to narrow this knowledge gap by thoroughly analyzing the composition of various plant protein ingredients used as emulsifiers (three soy protein ingredients and one pea protein ingredient) and correlating this to the oxidative stability of emulsions prepared with the corresponding protein fractions. The outcomes lay the groundwork for discussing the relative importance of the various components involved in the oxidative stability of food systems.

## 2 Materials and Methods

### 2.1 Materials

#### Protein Ingredients and Oil

Rapeseed oil (kindly supplied by Unilever, Wageningen, The Netherlands) was stripped from impurities and tocopherols using alumina powder (MP EcoChrome ALUMINA N, Activity: Super I, Biomedicals) according to a previously described protocol by Berton et al.^11^ Pea protein isolate (PPI) was obtained from Roquette (NUTRALYS S85F, purity 78%; N × 5.6^12^). Soy protein isolates (SPI) Supro Ex 37 IP (SPI-37) and Supro 772LN IP (SPI-LN) (International Flavors & Fragrances Inc., New York, USA), and soy protein concentrate (SPC) Arcon SJ (SPC-SJ) (ADM, Chicago, USA) were kindly supplied by Danone (Utrecht, The Netherlands) and Unilever (Wageningen, The Netherlands).

#### Materials to Measure Physical and Oxidative Stability

Sodium phosphate dibasic, sodium phosphate monobasic, chloroform, sodium azide (bactericide), butanol, ethyl acetate, chloroform, 2,4-dinitrophenylhydrazine (DNPH), ammonium thiocyanate (NH_4_SCN), guanidine hydrochloride (GuCl), trichloroacetic acid (TCA) 40%, 0.144 M iron(II) sulfate heptahydrate (FeSO_4_·7H_2_O), 0.132 barium chloride (BaCl_2_), ascorbic acid, and a bicinchoninic acid (BCA) kit (BCA1-1 KT) were purchased from Sigma-Aldrich (Zwijndrecht, The Netherlands). Hydrochloric acid (2 M) was obtained from Merck, Germany. 2,2,4-Trimethylpentane (isooctane), methanol and 2-propanol were obtained from Alfa Aesar (Kandel, Germany) and Actu-All Chemicals (Oss, The Netherlands), respectively. Deuterated chloroform (CDCl_3_) with 0.03% tetramethylsilane (TMS), deuterated dimethyl sulfoxide (DMSO-*d*_6_), and deuterated 4 Å mol sieves were purchased from Euriso-top (Saint-Aubin, France). Ultrapure water came from a Milli-Q system (Millipore Corporation, Billerica, Massachusetts, USA) and was used throughout the experiments.

#### Additional Materials for the Chemical Composition Measurement

Sodium cholate hydrate, disodium ethylenediaminetetraacetic acid (Na_2_EDTA·2H_2_O), tris(hydroxymethyl)aminomethane (TRIS), trisodium trimetaphosphate (TMP), phytic acid, gallic acid, and Folin–Ciocalteu reagent were purchased from Sigma-Aldrich (Zwijndrecht, The Netherlands). Deuterium oxide (D_2_O) was purchased from VWR international B.V. (Amsterdam, The Netherlands) and sodium carbonate (Na_2_CO_3_) was purchased from Thermo Scientific Chemicals (Waltham, MA, USA). Inositol standard was purchased from BDH Chemicals Ltd. (Poole, England) and sulfuric acid (H_2_SO_4_) from Merck KGaA (Darmstadt, Germany).

### 2.2 Methods

#### 2.2.1 Preparation of the Water-Soluble Protein Fraction

An aqueous dispersion of pea or soy protein ingredients was prepared as previously described by Hinderink et al.^13^ Briefly, a 6 wt % (isolates) or a 10 wt % (concentrate) suspension was prepared in 10 mM phosphate buffer (pH 7.0) and allowed to hydrate for at least 48 h under stirring at 4 °C. The soluble protein fraction was obtained by centrifuging the dispersion (16,000*g*, 20 °C, 30 min) and collecting the supernatant, which was in turn centrifuged again under the same conditions. The protein concentration in the supernatant was determined with the Dumas method,^14^ applying a nitrogen-to-protein conversion factor of 5.6 (PPI),^12^ 5.7 (SPI) or 5.38 (SPC).^15,16^ The supernatant, further referred to as protein solution and abbreviated as sPPI/sSPI/sSPC was then diluted to a protein concentration of 1.11 wt %, leading to a concentration of 1 wt % in the final emulsion.

#### 2.2.2 Compositional Analysis of the Protein Solutions

##### 2.2.2.1 Lipids

The total lipid content of the sPPI/sSPI/sSPC solutions was determined by an adaptation of the method described by Bligh and Dyer.^17^ A chloroform/methanol (2:1) extraction solvent was added to the protein solution (sample) in a solvent-to-sample volume ratio of 10:1 in a separation funnel. The mixture was shaken, and a 0.73 wt % NaCl solution was added to obtain a ratio of 4:1:1.5 (solvent:sample:NaCl solution). The obtained mixture was shaken 60 times with a degassing step every 20 steps. The separation funnels were placed for at least 24 h in a cold room at 4 °C to let the phases separate. The bottom chloroform phase was collected in a weighed flask, and chloroform (50% of the initial chloroform–methanol volume) was added to the methanol phase in the separation funnel. The funnel was again shaken 3 × 20 times before letting the mixture phase-separate again. The chloroform phase was again collected. The addition of chloroform to the methanol phase and the collection of the chloroform phase was repeated one more time. All chloroform phases were pooled, chloroform was evaporated under nitrogen flush at 25 °C and the final weight of the flasks with the extracted lipids was recorded. The lipid content was expressed in wt % of the protein solution.

The lipid composition was determined using ^1^H NMR with previously assigned integral regions and formulas.^18^ The extracted lipids (≤150 μL) were dissolved in 5:1 CDCl_3_:DMSO-*d*_6_ and transferred to a 5 mm NMR tube. Single pulse ^1^H NMR spectra were recorded on a 600 MHz (14.1 T) Bruker Avance III NMR spectrometer (Bruker BioSpin, Switzerland) equipped with a cryoprobe operating at 295 K. The phase correction, baseline correction, and integrations were performed automatically.

The phospholipid content was determined by using ^31^P NMR as previously described by Mayar et al.^19^ Briefly, a buffer solution was prepared containing 10% D_2_O with 120 g/L sodium cholate hydrate, 10 g/L disodium EDTA dihydrate, 0.25 g/L trimetaphosphate, and 10 g/L TRIS. The pH was adjusted to 7.5 with HCl 5 M. The freeze-dried powders of sPPI/sSPI/sSPC (this time, prepared in 10 mM NaCl instead of 10 mM phosphate buffer; Section 2.2.1) were hydrated in the buffer solution (15 mg/mL), mixed under head-overtail rotation for 1 h at 20 °C, and sonicated for 45 min at RT (no temperature control). The suspension was centrifuged at 5000*g* for 20 min (20 °C) and the supernatant was analyzed by ^31^P NMR. The spectra were recorded on a 700 MHz (16.4 T) Bruker Avance III HD NMR (Bruker BioSpin, Switzerland) equipped with a 10 mm BBO probe. The standard Bruker (zg) pulse program was used to record 512 scans at 300 K with 32,768 increments, a spectral width of 60 ppm, and an offset of 0 ppm. Phospholipids were quantified using trimetaphosphate as an internal standard. Spectra were processed by using an exponential window function with a 1 Hz line broadening, and signals were integrated in TopSpin v4.1.4 (Bruker BioSpin, Switzerland). Phospholipid signals were assigned by using standards and literature.^19^

Two independent samples were prepared and analyzed for each protein solution.

##### 2.2.2.2 Elemental Analysis

The elemental analysis was outsourced externally. Pea and soy protein ingredients (0.5–1 g) were mixed in a quartz tube with 65% nitric acid and digested in an Ultrawave microwave. The digest was diluted with water. Elements expected to be present at low concentrations in this solution (e.g., Fe, Cu) were assessed with an Agilent 8800 QQQ inductively coupled plasma mass spectrometer (ICP-MS). Elements expected at higher concentrations were determined with ICP-OES (PerkinElmer Optima 700DV). For quantification, calibration solutions in nitric acid were used. Results are reported as averages of duplicate measurements. Typical errors are <5% for ICP-OES and <10% for ICP-MS.

##### 2.2.2.3 Phytic Acid

Phytic acid content was analyzed using ^31^P NMR (along with phospholipids). Identification was performed by utilizing trimetaphosphate as a standard, and quantification was based on the signal at 1.23 ppm that exhibited the highest spectral isolation. This particular signal contained two identical phosphorus nuclei for each fully phosphorylated phytic acid molecule. The signals at 2.19, 1.64, 1.47, and 1.23 ppm exhibited a 1:2:2:1, respectively. To ensure precision and accuracy in the quantification process, each sample was spiked with 1 mg of a phytic acid standard. The relative standard deviation was lower than 10% and the recovery was around 97%. Complete phosphorylation was observed for phytic acid.

##### 2.2.2.4 Ashes

The ash content was determined by following the AACC method (08.01). Briefly, the weight of residual ashes was recorded after putting ∼20 g (exact weight recorded) of the samples for 8 h at 550 °C in an ashing furnace (Carbolite Gero, Sheffield, UK). The residue was defined as “ash” and was expressed in weight percentage of the protein solution.

##### 2.2.2.5 Carbohydrates

Neutral sugar composition was determined after prehydrolysis of 9–13 mg material with 72% (w/w) H_2_SO_4_ (1 h, 30 °C) followed by hydrolysis with 1 M H_2_SO_4_ (3 h, 100 °C) as described by Jermendi et al.^20^ Monosaccharides were derivatized to their alditol acetates using inositol as internal standard, and measured using gas chromatography coupled to flame ionization detector (GC-FID).^21^ Galacturonic acid content was determined by an automated colorimetric m-hydroxydiphenyl assay using an autoanalyzer (Skalar Analytical BV, Breda, The Netherlands).^22^

##### 2.2.2.6 Polyphenols

The concentration of free phenolic compounds (i.e., not covalently bound to proteins) was determined after an extraction step, for which ∼40 mg of freeze-dried sample was first weighed into 2 mL Eppendorf tubes (±0.5 mg). Methanol was added in a sample:solvent ratio of 1:20 (w/w). The suspension was sonicated for 15 min and centrifuged for 15 min at 15,000*g* (RT). The supernatant was divided into three weighed glass vials (3 × 240 μL), the extraction (solvent addition, sonication, and centrifugation) was repeated, and the supernatant was again divided into three glass vials. The extracts were dried under nitrogen flow in the dark, and the masses of the extracts were recorded. Afterward, the phenolic content was determined by using the Folin–Ciocalteu assay. The samples were prepared by first redissolving the extracts in 1 mL methanol, vortexing (1 min), and sonication (30 min) at RT. Insoluble components were removed by centrifugation at 15,000*g* for 15 min (RT). A volume (50 μL) of unknown sample or gallic acid standard solution was mixed with ultrapure water (750 μL) and the Folin–Ciocalteu reagent (50 μL) in an Eppendorf tube and vortexed for 3 min at RT. After addition of a saturated Na_2_CO_3_ solution (300 μL), the dispersion was shortly vortexed again and incubated for 60 min in the dark (RT). The absorbance of the supernatant (centrifugation for 2 min at 21,100*g* at RT) was measured at 765 nm. The phenolic compound concentration was calculated by using the gallic acid standard curve and expressed in wt % gallic acid equivalent.

##### 2.2.2.7 Protein Composition

The SDS-PAGE assays for emulsions were run under reducing conditions as described by Hinderink at al. with one additional washing step of the cream phase.^13^ Gels were scanned and analyzed using a calibrated densitometer (GS-900, Biorad laboratories, USA) and Image Lab software (Bio-Rad laboratories, USA).

#### 2.2.3 Preparation of the Emulsion

A coarse emulsion was prepared by mixing 10 wt % stripped rapeseed oil with an aqueous phase (1 wt % sPPI/sSPI/sSPC) using a high-speed blender (S18N-19G, Ultraturrax R, IKA-Werke GmbH & Co., Staufen, Germany) at 11,000 rpm for 1 min. The coarse emulsion was passed through a high-pressure homogenizer (M-110Y Microfluidizer, Microfluidics, Massachusetts, USA) equipped with a F-12Y interaction chamber at 400 bar to obtain the final emulsion after five passes. The coil of the system was cooled by ice water to prevent heating up of the emulsion during preparation. The sodium azide (0.02 wt %) was added and emulsions were stored in 20 mL vials (6 mL per vial), and horizontally rotated (3 rpm) in an oven at 40 °C, in the dark. Samples were taken at day 0, 1, 3, 7, and 14 for physical and oxidative stability analysis; two emulsions were prepared independently, for each formulation.

#### 2.2.4 Physical Stability

##### 2.2.4.1 Droplet Size Distribution

The emulsion droplet size distribution was measured by static light scattering (Mastersizer 3000, Malvern Instruments Ltd.; Worcestershire, UK) using the refractive indexes for water (1.330) and rapeseed oil (1.473) and an absorption index of 0.01. All emulsions were measured as such and after dilution in a 1 wt % SDS solution (1:1 v/v) to distinguish between the apparent droplet size distribution including possibly aggregated droplets and the actual size distribution of individual droplets. The average droplet size is reported as the Sauter mean diameter (*d*_3,2_). Each result is the mean of at least two independent emulsion samples, each taken from two independently prepared emulsions. (The average of five measurements is taken as the result for each sample).

##### 2.2.4.2 Emulsion Morphology

The morphology of the emulsions was visualized using light microscopy (Axioscope, Zeiss, Germany) at 40× magnification, without dilution.

##### 2.2.4.3 Zeta Potential

The zeta potential was determined by measuring the electrophoretic mobility of droplets via laser Doppler electrophoresis and phase analysis light scattering (PALS) using a Zetasizer Nano ZS (Malvern Instruments Ltd.; Worcestershire, UK). The zeta potential was calculated by using the Smoluchowski model with refractive indices of 1.330 and 1.473 for water and rapeseed oil, respectively. Samples were 101-fold diluted with ultrapure water and measured after 3 min of equilibration at room temperature with three measurements per sample. The reported zeta potentials are the average values for two independent emulsions, which were each measured three times.

#### 2.2.5 Oxidative Stability

##### 2.2.5.1 Lipid Oxidation

Lipid oxidation in the emulsions was measured with ^1^H NMR according to the method of Merkx et al.^23^ Prior to the measurement, oil was extracted by adding isooctane:isopropanol (3:2) to the emulsion (4:1 v/v), vortexing 3 times for 20 s each, and centrifuging for 8 min at 4700 rpm. The iso-octane layer was collected, and the solvent was evaporated under nitrogen flow (Reacti-Therm III, Thermo Fisher Scientific, USA) at 25 °C. Each data point is the average of at least two individual emulsions, of which each was measured two times. Standard deviations are calculated based on all measurements (*n* = 4) of the different replicates combined.

##### 2.2.5.2 Protein Oxidation

Protein oxidation was determined by measuring the carbonyl content according to the DNPH method described by Levine and co-workers.^24,25^ Proteins were precipitated from the emulsions using isopropanol (1:10) followed by a centrifugation step at 15,000*g* (5 min, RT) to obtain the protein pellet. The protein pellets were dispersed in either 500 μL of 10-mM DNPH in HCL 2 N, or only in HCL 2 N (blanks). After incubation for 60 min in the dark, proteins were precipitated again with 500 mL of 400 g/L trichloroacetic acid solution for 10 min on ice. The dispersion was centrifuged again at 15,000*g* for 5 min at RT and the pellet was washed with 1 mL of ethanol/ethyl acetate 1/1 v/v (2×), with 1 mL of 2-propanol (1×), and finally dissolved in 1 mL of guanidine hydrochloride (GuCl) 6 M at 37 °C. Another centrifugation step carried out under the same conditions removed the insoluble fraction, if any, and the absorbance of the supernatant was measured at 370 nm. A molar absorption coefficient of 22,000 M^–1^ cm^–1^ was used to calculate the protein-bound carbonyl content. The results were expressed in millimolar carbonyl per kilogram of soluble protein. The soluble protein concentration in the final supernatant was determined by the BCA assay using sPPI/sSPI/sSPC solutions of known concentration as calibration solutions.^26^ For the BCA assay, the GuCl concentration in the unknown samples and in the calibration solutions was adjusted to 2 M (maximum allowed concentration according to the supplier). Protein oxidation was determined in two emulsions that were independently prepared, and each was measured twice (*n* = 4).

#### 2.2.6 Statistical Analysis

The significance of concentration differences was determined with IBM SPSS statistics software with one-way ANOVA and posthoc with the Tukey HSD method to compare means.^27^ Significance was established with *p* < 0.05. A correlation matrix was produced using again IBM SPSS statistic software with the commonly used Pearson correlation coefficient.^28^

## 3 Results and Discussion

### 3.1 Chemical Composition of the Protein Solutions

**Figure 1:**
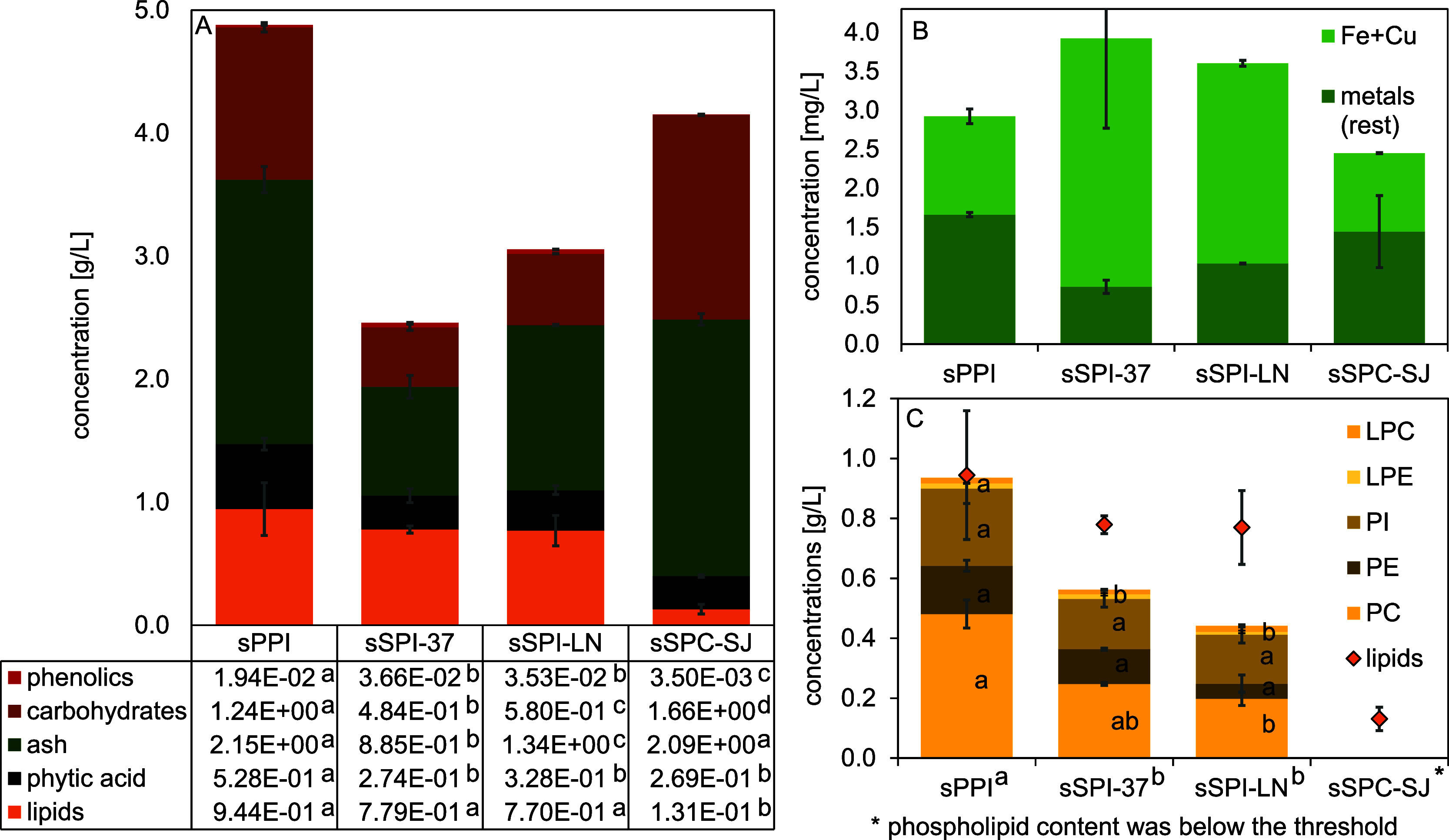
Content of the (A) main non-protein components, (B) iron, copper, and other metal ions, and (C) phospholipid composition (LPC: lysophosphatidylcholine; LPE: lysophosphatidylethanolamine PI: phosphatidylinositol; PE: phosphatidylethanolamine; PC: phosphatidylcholine) together with the total lipid content in the soluble fraction (prefix ‘s’) of the different ingredients (PPI: pea protein isolate; SPI: soy protein isolate; SPC: soy protein concentrate). The error bars show the standard deviations of at least two individual measurements. Small letters indicate significant differences between the protein solutions (*p* < 0.05).

The soluble fractions of all protein ingredient dispersions were collected after centrifugation and analyzed for their chemical composition. Concentrations are given for soluble fractions adjusted to 1 wt % protein (Figure 1).

**Figure 2:**
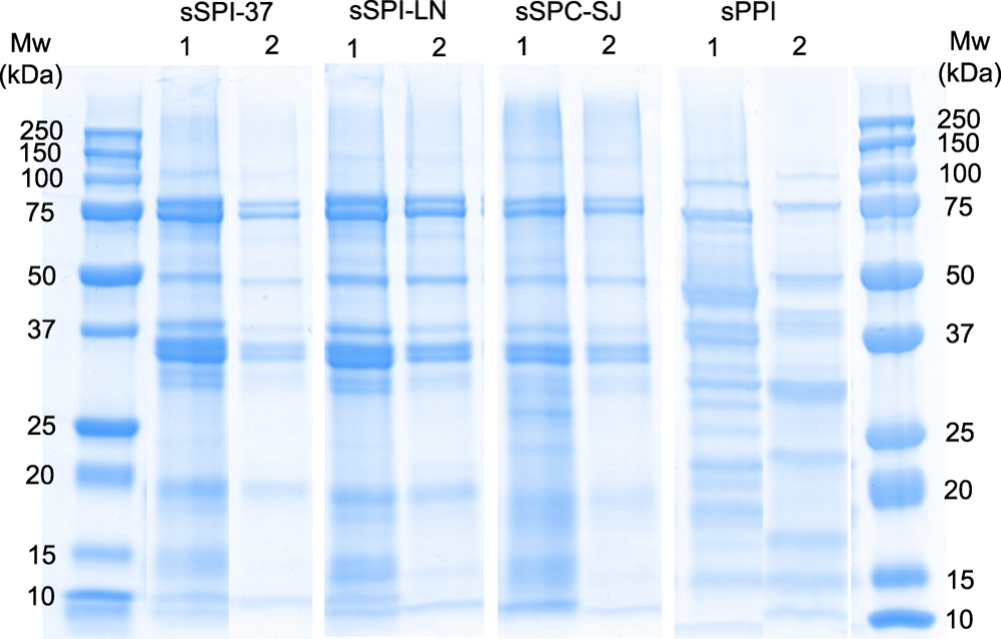
SDS-PAGE profiles under reducing conditions of the soy and pea protein stabilized emulsions (prefix ‘s’: soluble fraction; PPI: pea protein isolate; SPI: soy protein isolate; SPC: soy protein concentrate) with (1) the cream and (2) the aqueous phase. The first and last lane correspond to the molecular weight markers.

The types of proteins present in the different soluble fractions and their distribution in the aqueous and creamed phases of the emulsions were determined by SDS-PAGE (Figure 2). As expected, all soy protein solutions contained both glycinin (basic and acidic) and β-conglycinin subunits (α′, α, and β) (Table S2, Supporting Information)^29,30^ with the ratio between glycinin and β-conglycinin being very similar for the isolates and concentrate (Figure S2, Supporting Information). For the SPI-37 emulsion, the relative amount of the glycinin subunits (i.e., acidic) was higher compared to the two other soy protein emulsions where a relatively higher amount of β-conclycinin (i.e., α′) was adsorbed at the interface. The molecular weights of the glycinin subunits slightly varied among the different ingredients. The pea protein isolate contained both vicilin and legumin subunits,^31,32^ with a similar protein composition for the creamed and aqueous phases of the emulsions, which is in line with Hinderink et al.^13^

Regarding the non-protein components, the soluble fraction of protein isolates contained significantly more lipids than that of the concentrate (Figure 1), which is counterintuitive. Lipids are known to hinder protein ingredient production from soybeans and are therefore removed early in the process by defatting, which explains the relatively low level in the soy concentrate.^33−35^ For soy protein isolates, the higher concentrations may arise from the formation of lipid–protein complexes which could be enhanced by conformational changes of the proteins at high pH.^36,37^ Keuleyan et al. found a higher lipid content in pea protein isolate compared to pea protein concentrate.^10^ In the former case, the lipid content was substantially higher than the normal levels in peas, suggesting that isoelectric wet fractionation processes result in an accumulation of endogenous lipids in the final ingredient when no defatting step is included.

When digging deeper into the type of lipids involved, the sPPI was significantly higher in phospholipids compared with the soy protein isolate solutions (Figure 1C). More in general, the phospholipids were the most prominent lipid components present in those isolate samples (Figure 1C).^38,39^ For the concentrate, this could not be determined because the phospholipid concentration was below the detection threshold of the applied method. The values that we report here are in general higher than those found in the literature. Keuleyan et al. reported that ∼50 wt % of the lipids in pea and lupin protein isolates were phospholipids and the other ∼50 wt % neutral lipids, whereas for lupin concentrate the phospholipids accounted for ∼25 wt %.^10^ It is good to point out that Keuleyan et al.^10^ analyzed the whole protein ingredients, whereas the present work focused on the soluble fractions only. This could indicate that the lipids that end up in the soluble fraction are preferentially phospholipids (polar lipids), whereas neutral lipids (triglycerides) are less likely to be extracted.

The PPI solution contained 0.53 g/L phytic acid, which is significantly higher compared to the soy protein solutions, which may be the result of inherent content differences in the seeds, and/or of a more extensive removal of this antinutritional component during the fractionation process.^9^ The ash and carbohydrate concentrations were significantly higher in sSPC and sPPI compared to both sSPI samples, which is in line with literature where higher concentrations of carbohydrates were reported for concentrates compared to isolates, and for pea compared to soybean.^9,40^ Both sSPIs contained significantly more free phenolics compared with sPPI and sSPC.

All protein solutions contained considerable amounts of metal ions that are on the order of micrograms per liter. Figure 1B shows that the content in iron and copper was the highest for sSPIs, whereas sPPI and sSPC contained relatively more metal ions. The metal content can vary a lot between crops but also cultivars,^41^ and will be affected by the process steps carried out to obtain the protein ingredients. Especially pH shifts can lead to the complexation of cations by negatively charged amino acid side groups (pH > isoelectric point), as would be the case during wet fractionation that is used to obtain protein isolate.^42^

### 3.2 Physical Stability of Emulsions

**Figure 3:**
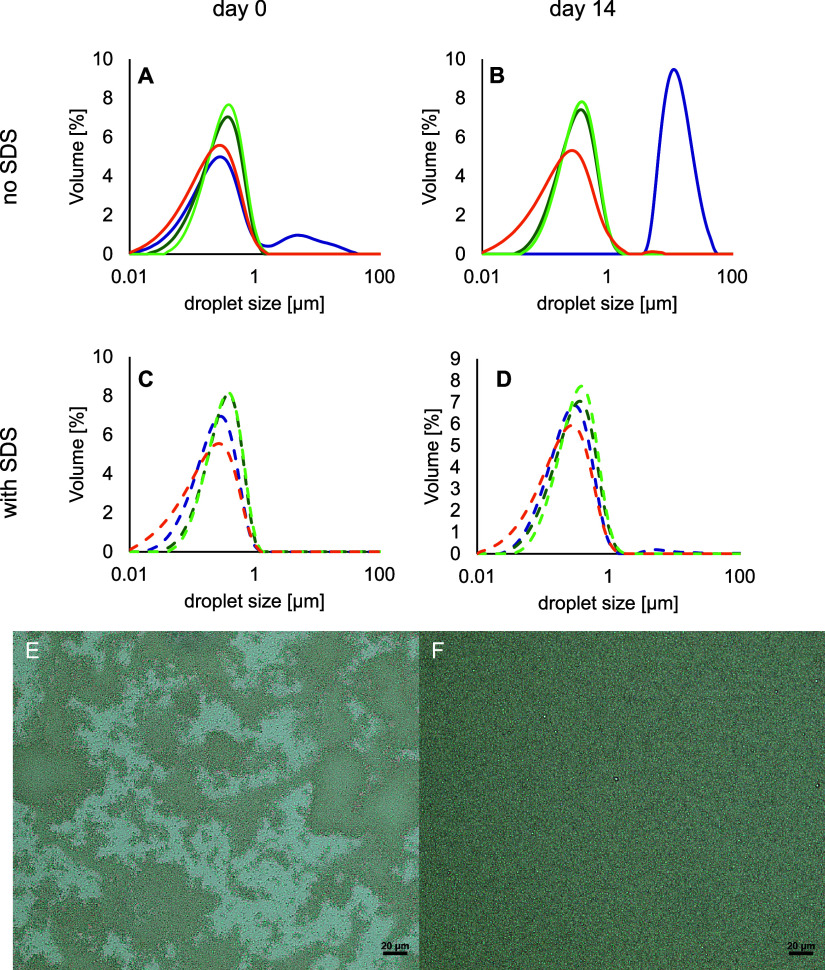
Droplet size distribution of the emulsions at day 0 (A, C) and day 14 (B, D) stabilized with soluble fractions adjusted to 1 wt % protein for pea protein isolate (sPPI: dark blue), soy protein isolates (sSPI-37: dark green and sSPI-LN: light green), or soy protein concentrate (sSPC-SJ: orange). The distribution was measured as such (solid lines) or after 2-fold dilution of the emulsions with 1 wt % SDS (dashed lines) to assess possible flocculation (*n* = 5). The displayed data are representative; similar trends were obtained for two independent replicates. Light microscopy images of emulsions stabilized by sPPI (E) and sSPI-37 (F) after 14 days of incubation. The scale bars represent 20 μm. Very similar images to the one shown in panel (F) were recorded for the 14-day incubated emulsions prepared with sSPI-LN and sSPC-SJ (Figure S3, Supporting Information).

The physical stability of emulsions prepared with the soluble fractions of the different plant protein ingredients was studied by measuring the droplet size distribution over time. The full droplet size distributions are shown in Figure 3A–D; the average droplet size (*d*_3,2_), including those after dilution in SDS, to test for flocculation, and the ζ-potential of the droplets, can be found in Table 1.

**Table 1:** Average Droplet Size (*d*_3,2_) of the Emulsions Freshly Prepared, after 14 Days of Incubation, and Average Zeta Potential^a^

	*d*_3,2_ [μm]		
	day 0	day 14	ζ potential [mV]
sPPI	0.17 (0.17)	12.5 (0.20)	–39.35 ± 1.45
sSPI-37	0.21 (0.25)	0.25 (0.22)	–48.06 ± 1.99
sSPI-LN	0.26 (0.27)	0.28 (0.27)	–49.69 ± 2.65
sSPC-SJ	0.12 (0.11)	0.12 (0.13)	–44.01 ± 2.83

a Prefix ‘s’: soluble fraction; PPI: pea protein isolate; SPI: soy protein isolate; SPC: soy protein concentrate. Numbers in brackets give the average droplet size when 2× diluted in 1 wt % SDS.

The droplet size distributions of the emulsions stabilized by soy protein solutions were very similar for the freshly prepared and 14-day incubated emulsions, with no change when diluted in 1 wt % SDS. This indicates that no flocculation nor coalescence occurred in those emulsions, which is well in line with the strong negative surface charge of the droplets (see Table 1, and the optical microscopy pictures; Figure 3E,F). From the protein composition at the interface a higher physical stability would have been expected for the emulsions with a higher relative amount of β-conglycinin.^43^ However, this could not be confirmed in this work. In contrast, the emulsion stabilized by the pea protein solution showed a bimodal distribution, which is due to flocculation as we conclude from the fact that the original droplet size distribution is found back when diluting in SDS. The zeta-potential of the sPPI-emulsions was only (slightly) less negative than for the soy-emulsions and is not expected to have been the cause for this difference.^44^ This is also very obvious from the microscopic pictures (Figure 3E,F). A possible explanation could be an effect that is inherent to differences in protein structures, e.g., *N*-glycosylation sites of soybean 7S globulin which might enhance emulsifying ability.^45^ In addition, Can Karaca et al. reported a higher surface hydrophobicity for pea protein isolate compared to soy protein isolate.^46^ A high hydrophobicity leads to attractive forces being more prominent and thereby to proteins forming aggregates or bridges between droplets when adsorbed at the interface.^12,44^ Alternatively, the presence of high level of phosphatidylcholine at the interface may have led to patchy surfaces that would make the droplets more prone to aggregation.^47,48^

### 3.3 Oxidative Stability of Emulsions

**Figure 4:**
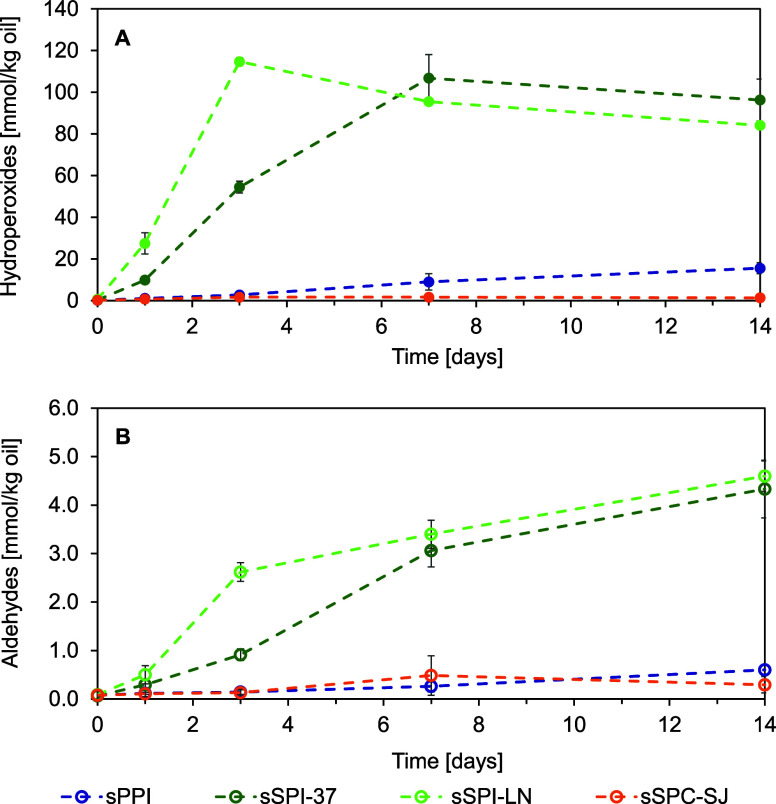
(A) Hydroperoxide and (B) aldehyde concentration in emulsions (prefix ‘s’: soluble fraction; PPI: pea protein isolate; SPI: soy protein isolate; SPC: soy protein concentrate) for 14 days of storage (40 °C; horizontally rotating in the dark). The error bars show the standard deviation of two independent duplicates and measurements (*n* ≤ 2; *i* ≤ 2). The lines are drawn to guide the eye.

Lipid oxidation was assessed by measuring the primary (hydroperoxide) and secondary oxidation products (aldehydes) at regular time points during incubation (Figure 4). Over 14 days of storage, emulsions either oxidized very fast (sSPI-emulsions), or barely (sPPI-emulsion) to not (sSPC-SJ-emulsion). Thus, while the soy- and pea protein-stabilized emulsions investigated in this work all displayed good physical stability, they were found to largely differ when considering their propensity to lipid oxidation. The protein solutions used to stabilize the emulsions all contained the same protein concentration (1 wt % as final concentration in the emulsions), and the soy protein solutions had similar protein profiles (Figure 2), but rather different non-protein component profiles (Figure 1). In addition, proteins in isolates and concentrates differ in their oxidative state due to the production conditions, which may affect the oxidative emulsion stability.^9^

To try to rationalize and deconvolute the effects of the multitude of factors at play, a correlation matrix was prepared containing the chemical composition of the soluble plant protein solutions and oxidation data of the emulsions (hydroperoxide concentration at day 3 and 7, and aldehyde concentration at day 3) (Table S2). The key factors are summarized in Table 2, focusing on the hydroperoxide concentration at day 7: only correlation values (*r*) exceeding an absolute value of 0.8 are shown.

**Table 2:** Overview of the Most Relevant Components for Lipid Oxidation (Correlation Coefficient > ±0.8)

	**sPPI**	**sSPI-37**	**sSPI-LN**	**sSPC-SJ**	**correlation value (***r***) HPX (day 7)**
Fe + Cu[mg/L]	1.26	3.15	2.57	1.01	0.978^b^
minerals [mg/L]	1158	441	713	950	–0.900^a^
ash [g/L]	2.15 ± 0.11	0.89 ± 0.24	1.34 ± 0.01	2.09 ± 0.10	–0.968^b^
phenolics[mg/L]	19.35 ± 0.33	36.55 ± 3.23	35.28 ± 2.22	3.50 ± 0.18	0.927^a^
protein oxidation [μmol carbonyls/g soluble protein]	2.94 ± 0.65	7.10 ± 1.82	6.22 ± 2.08	4.51 ± 1.08	0.914^a^
ζ-potential [mV]	–39.35 ± 1.45	–48.06 ± 1.99	–49.69 ± 2.65	–44.01 ± 2.83	0.850

a Significant at the 0.05 level.

b Significant at the 0.01 level; HPX: hydroperoxide.

A high concentration of iron and copper was correlated with a higher hydroperoxide concentration (*r* = 0.98; *p* < 0.01), which is not surprising since it is well-known that these metal ions can act as prooxidants by decomposing pre-existing traces of lipid hydroperoxides into peroxyl or alkoxyl radicals, and hydrogen peroxide via the Fenton reaction, to form highly reactive hydroxyl radicals.^49−51^ Metal ions represent only a very small fraction of the total ashes (<0.5% of total metal content and <0.4% of iron and copper content combined). This implies that other minerals are present in substantial amounts, with sPPI and sSPC having the highest mineral as well as ash concentration (Table 2). Minerals such as sodium or potassium have been reported to shield the (negative) charge of proteins and therefore the concentration of pro-oxidant metal ions near the protein-covered interfaces may be lower.^52^ This could explain the negative correlation between the total mineral content and hydroperoxides formed (*r* = −0.90; *p* < 0.05), although it is good to point out that all emulsions have a considerable negative charge, and other effects are expected to also play a role.

The content in phenolic compounds, as found in sSPI samples, was positively correlated with lipid oxidation (*r* = 0.91/0.81), which is counterintuitive since phenolic compounds are generally known to act as antioxidants. However, at high concentrations and in the presence of iron, it is known that the formation of reactive oxygen species can take place via redox cycling and initiation of the Fenton reaction, thereby promoting lipid oxidation.^53−55^ It should be mentioned that we measured only free phenolics, which implies that possible covalent conjugates with proteins are not taken into account. When conjugated, they have generally been reported to increase the oxidative stability of emulsions compared to noncovalent complexes.^56^

Next to non-protein components, the initial level of protein oxidation was positively correlated with lipid oxidation in emulsions (*r* = 0.91; *p* < 0.05 for hydroperoxides). Radical-mediated protein and lipid oxidation can mutually promote each other.^5,57−59^ Therefore, it may be expected that an initial level of protein oxidation, as a result of the protein fractionation process used (i.e., defatting and wet processing), would be a driver for subsequent lipid oxidation in emulsions formulated with those ingredients, although systematic experimental evidence on this matter is still missing.^9^ The protein-bound carbonyl content in the fresh emulsions (between 2.94 and 7.01 μmol/g soluble protein) was consistent with values from literature for commercial plant protein ingredients (∼3–20 mmol/kg),^60,61^ which clearly points to an aspect of protein materials that is generally overlooked, but that could turn out to be highly relevant for their use.

To summarize, our analysis connecting compositional data of protein-rich plant extracts with lipid oxidation markers in oil-in-water emulsions revealed that high levels of soluble metal ions (iron and copper), phenolic compounds, and initial protein oxidation in the extract were associated with reduced oxidative stability of the emulsions. On the other hand, protein extracts with high ash content seemed to improve the oxidative stability of the emulsions. Future research should aim to deepen understanding of these relationships, possibly by experimenting with various ratios of non-protein components. Moreover, considering the complex and variable composition of commercial plant protein ingredients, it would be very advisible to everyone working in this field to pay close attention to the source and the composition of alternative protein ingredients, including their oxidative status, and to comprehensively report these details. This approach is crucial to accelerate progress required for a successful transition toward plant-protein-rich diets based on healthy, nutritious, tasty, and stable products.

## Supporting Information Available

Figure S1: Fatty acid composition of all lipids in the different ‘soluble’ protein solutions. Table S1: Molecular weight distribution of soy and pea protein subunits. Figure S2: Interfacial composition of the soy protein emulsions. Figure S3: Light microscopy images of emulsions stabilized by sSPI-LN and sSPC-SJ after 14 days of incubation. Table S2: Correlation matrix (PDF)

## Notes

K.M.: Conceptualization, data curation, formal analysis, investigation, methodology, visualization, and writing–original draft; S.S. and K.S.: conceptualization, investigation, methodology, supervision, and writing–review and editing; C.B.-C.: Conceptualization, investigation, methodology, writing–review and editing, supervision, and funding acquisition.

## Abbreviations Used

(s)SPI: (soluble fraction of) soy protein isolate
(s)SPC: (soluble fraction of) soy protein concentrate
(s)PPI: (soluble fraction of) pea protein isolate
