# Supplementary material for: Effect of Nonprotein Components for Lipid Oxidation in Emulsions Stabilized by Plant Protein Extracts
Source: ACS Food Sci Technol. 2024 Mar 22;4(4):926–34. doi: 10.1021/acsfoodscitech.3c00691 (PMC11036399; doi:10.1021/acsfoodscitech.3c00691)
Supplement: Supplementary file 1 — fs3c00691_si_001.pdf [file fs3c00691_si_001.pdf]

Effect of non-protein components for lipid oxidation in emulsions stabilized by plant protein extracts

Katharina Münch[a,*], Simeon Stoyanov[b,c], Karin Schroën[a], Claire Berton-Carabin[a,d]

[a] 6708 WG Wageningen, Gelderland, The Netherlands

[b] 6708 WE Wageningen, Gelderland, The Netherlands

[c] 10 Dover Drive, Singapore 138683

[d] 44300 Nantes, France

Summary of the number of pages: 5

Figures:

Page 1: Figure S1: Fatty acid composition of all lipids in the different 'soluble' protein solutions.

Page 3: Figure S2: Interfacial composition of the emulsions stabilised by the soluble fraction of soy protein isolate (SPI) or concentrate (SPC) at day 0 determined with SDS-PAGE densitometry. The ß-conglycinin subunits are shown in green shades and glycinin subunits in blue shades.

Page 4: Figure S3: Light microscopy images of emulsions stabilised by the soluble fraction of soy protein isolate (SPI-LN; A) and soy protein concentrate (SPC-SJ; F) after 14 days of incubation. The scale bar represents 20 µm.

Tables:

Page 2: Table S1: Molecular weight distribution of soy and pea protein subunits.

Page 5: Table S2: Correlation matrix.

Supporting data.

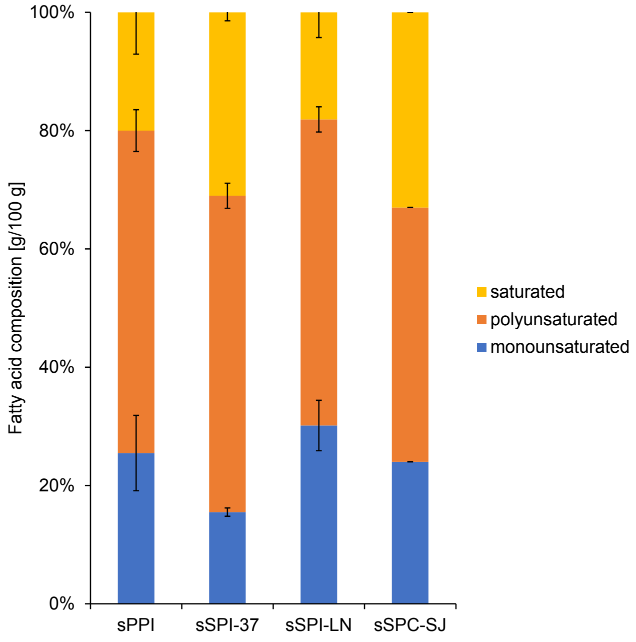

Figure S1: Fatty acid composition of all lipids in the different 'soluble' protein solutions.

Table S1: Molecular weight distribution of soy and pea protein subunits.

Mw (kDa)	Soy proteins[i]	Pea proteins[ii]
>91	lipoxygenase	lipoxygenase
73-82	conglycinin (α’)	convicilin
67-73	conglycinin (α/α’)	
55-67	conglycinin (α)	
49-55	conglycinin	
44-49	conglycinin (γ/ß)	Vicilin major subunit
34-44	glycinin (acidic)	α-legumin
26-34		Vicilin minor subunit
22-26	glycinin (not defined)	ß-legumin
14.4-22	glycinin (basic)	Vicilin minor subunit
10		Protease inhibitor

i)[29,30]; ii)[31]

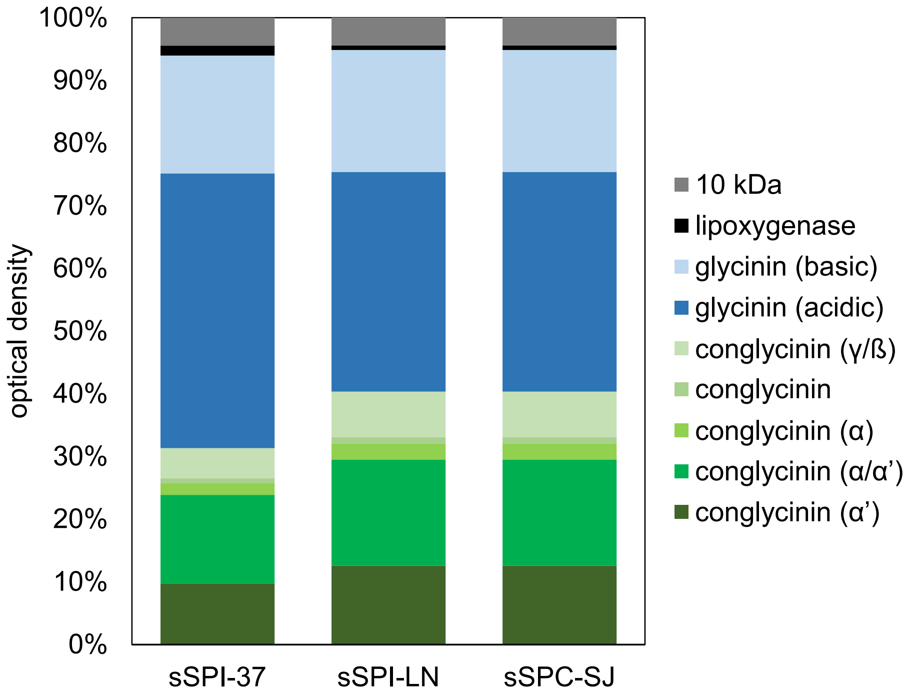

Figure S2: Interfacial composition of the emulsions stabilised by the soluble fraction of soy protein isolate (SPI) or concentrate (SPC) at day 0 determined with SDS-PAGE densitometry. The ß-conglycinin subunits are shown in green shades and glycinin subunits in blue shades.

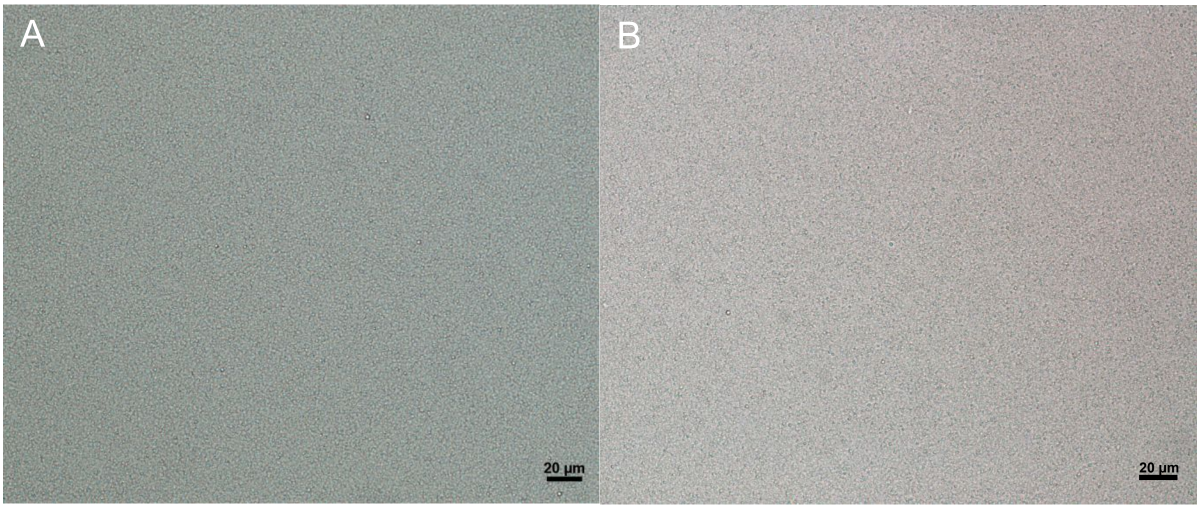

Figure S3: Light microscopy images of emulsions stabilised by the soluble fraction of soy protein isolate (SPI-LN; A) and soy protein concentrate (SPC-SJ; F) after 14 days of incubation. The scale bar represents 20 µm.

Table S2: Correlation matrix.

	Fe + Cu	Metal (total)	Phosphate	Minerals	Ash	Phytic acid	Monounsaturated	Polyunsaturated	Saturated	Lipids (total)	Carbohydrates	Phenolics	Protein oxidation	ζ-potential	HPX (day 3)	HPX (day 7)	Ald (day 3)	Ald (day 7)
**Fe + Cu**	1																	
**Metal (total)**	0.904*	1																
**Phosphate**	-0.364	-0.165	1															
**Minerals**	-0.916*	-0.732	0.702	1														
**Ash**	-0.965**	-0.765	0.495	0.960**	1													
**Phytic acid**	-0.396	-0.331	0.919*	0.667	0.438	1												
**Monounsaturated**	0.209	0.374	0.818	0.194	-0.038	0.657	1											
**Polyunsaturated**	0.445	0.572	0.635	-0.069	-0.296	0.583	0.868	1										
**Saturated**	0.618	0.514	0.205	-0.413	-0.611	0.370	0.456	0.796	1									
**Lipids (total)**	0.461	0.552	0.631	-0.089	-0.328	0.597	0.876	0.995**	0.8165	1								
**Carbohydrates**	-0.693	-0.877	-0.314	0.370	0.502	-0.146	-0.722	-0.877	-0.654	-0.852	1							
**Phenolics**	0.857	0.720	0.033	-0.657	-0.827	0.018	0.555	0.634	0.698	0.682	-0.683	1						
**Protein oxidation**	0.911*	0.746	-0.687	-0.985**	-0.939*	-0.707	-0.149	0.038	0.311	0.059	-0.372	0.676	1					
**ζ-potential**	-0.752	-0.489	0.582	0.836	0.831	0.580	0.090	0.085	-0.168	0.028	0.140	-0.711	-0.890*	1				
**HPX (day 3)**	0.743	0.628	-0.124	-0.622	-0.709	-0.259	0.413	0.293	0.247	0.343	-0.469	0.866	0.718	-0.855	1			
**HPX (day 7)**	0.978**	0.828	-0.347	-0.900*	-0.968**	-0.370	0.235	0.397	0.575	0.431	-0.611	0.914*	0.914*	-0.850	0.848	1		
**Ald (day 3)**	0.633	0.529	-0.049	-0.505	-0.596	-0.202	0.444	0.251	0.148	0.304	-0.399	0.809	0.618	-0.810	0.988**	0.757	1	
**Ald (day 7)**	0.926*	0.756	-0.366	-0.869	-0.928*	-0.411	0.214	0.292	0.444	0.335	-0.513	0.901*	0.910*	-0.920*	0.919*	0.982**	0.851	1

HPX: hydroperoxide; Ald: aldehydes

* significant at the 0.05 level;** significant at the 0.01 level.
